# Comparison of acetic acid and ethanol sclerotherapy for simple renal cysts: clinical experience with 86 patients

**DOI:** 10.1186/s40064-016-1971-5

**Published:** 2016-03-09

**Authors:** Young Jun Cho, Ji Hoon Shin

**Affiliations:** Department of Radiology, Busan Paik Hospital, Inje University College of Medicine, 633-165 Gaegeum-dong, Busanjin-gu, Busan, 614-725 Korea; Department of Radiology, Asan Medical Center, Research Institute of Radiology, University of Ulsan College of Medicine, 388-1, Pungnap-2dong, Songpa-gu, Seoul, 138-736 Korea

**Keywords:** Simple renal cyst, Sclerotherapy, Acetic acid, Ethanol

## Abstract

**Background:**

To compare the efficacy and treatment session numbers of acetic acid to that of ethanol sclerotherapy for the treatment of simple renal cysts. Between February 2004 and June 2013, 86 patients with simple renal cysts underwent percutaneous aspiration and injection of 50 %-acetic-acid (42 cysts) and 95 %-ethanol (44 cysts). The patient demographics, volume reduction rate, number of treatment sessions, and complications were then analyzed.

**Results:**

The volume reduction rate was 94.1 ± 7.6 % in the 50 %-acetic acid group and 94.7 ± 11.7 % in the 95 %-ethanol group, and without a statistical difference. The rates of complete remission, partial remission, and no response were 57.1, 42.9 and 0 %, respectively, for the acetic acid group, and 70.5, 25.0, and 4.5 %, respectively, for the ethanol group. No statistical difference was observed between the two groups. Compared to the acetic acid group, the ethanol group had a higher number of treatment sessions, i.e. 1.10 ± 0.30 in the acetic acid group and 1.80 ± 0.79 in the ethanol group. Mild flank pain was a minor complication that occurred in both groups.

**Conclusions:**

Acetic acid seems to have equivalent sclerosing effects on simple renal cysts compared with those of ethanol despites of fewer treatment sessions.

## Background

Simple renal cysts are the most commonly detected type of renal mass and usually do not require treatment (Clayman et al. 1984). However, when large renal cysts are associated with flank or back pain, hypertension or renal calyceal or pelvic obstruction, they require treatment (Brown et al. 1995). Symptomatic renal cysts can be treated by a variety of surgical and interventional managements such as percutaneous aspiration with or without sclerotherapy (Amar and Das 1984; Agarwal et al. 2012; Zerem et al. 2008; el-Diasty et al. 1995). Surgical treatment and laparoscopic deroofing are more invasive, and thus necessitate general anesthesia with the accompanying potential operative morbidity and possible complications. Compared to other surgical treatment methods, percutaneous needle aspiration and sclerotherapy are less invasive (Agarwal et al. 2012). Although various sclerosants have been used, alcohol is the most commonly used sclerosing material for cyst ablation (Amar and Das 1984; Mohsen and Gomha 2005; Kwon et al. 2007; Choi et al. 2009; Egilmez et al. 2007). However, alcohol is related to potential complications such as pain, fever, a drunken state, shock, loss of consciousness, and femoral nerve injury (Kim et al. 2004; Ashraf et al. 2012).

Acetic acid has been used as the sclerosant that induces coagulation necrosis around the injection site in rat livers and in the treatment of small hepatocellular carcinomas <3 cm in humans (Ohnishi et al. 1994). A high long-term success rate, even at a low dose, was reported in one published study in the management of renal cysts (Yoo et al. 2008). There are, however, only two studies that compared the effectiveness of acetic acid and ethanol in the sclerotherapy of simple renal cysts (Cho et al. 2008; Seo et al. 2000). However, in these studies, there was no attempt to compare the treatment session of each sclerosant. Therefore, we compared the efficacy and treatment session numbers of 50 %-acetic acid and 95 %-ethanol sclerotherapy for the treatment of simple renal cysts.

## Methods

### Patients

Between February 2004 and June 2013, 86 patients with simple renal cysts underwent ultrasonography (US) or fluoroscopy-guided percutaneous aspiration and injection of 50 %-acetic acid (42 cysts) and 95 %-ethanol (44 cysts). Forty-nine patients were male and 37 were female. The average patient age was 61.1 years (range 14–84 years). Table 1 shows the baseline characteristics of the two groups. All patients underwent US or computed tomography (CT) before and after their sclerotherapy. The indications for interventional treatment were large cyst (>4 cm) (n = 49), enlarged cysts (n = 22), flank pain (n = 13), nausea (n = 1), and hydronephrosis (n = 1). The risk and benefits of and the alternatives to the procedure were explained to all patents, and informed consent for the procedure was obtained from all of the patients. This retrospective study was approved by our hospital’s institutional review board.

**Table 1:** Patients’ baseline characteristics

Variable	Overall	Group		
		Acetic acid	Ethanol	p value
Number (%)	86 (100.0)	42 (48.8)	44 (51.2)	
Age	61.1 ± 11.0	59.7 ± 13.1	62.3 ± 8.5	0.2721^a^
Sex				
Male	49 (57.0)	22 (52.4)	27 (61.4)	0.400^b^
Female	37 (43.0)	20 (47.6)	17 (38.6)	
Cyst location				
Right	36 (41.9)	19 (45.2)	17 (38.6)	0.535^b^
Left	50 (58.1)	23 (54.8)	27 (61.4)	
Pretreatment volume (ml)	206.7 ± 242.1	194.9 ± 211.2	217.9 ± 270.3	0.116^c^
Follow-up period (days)		326.7 ± 296.6	727.2 ± 854.2	0.258^a^

^a^Independent t test

^b^Chi square test

^c^Mann–Whitney U test

### Procedures and analysis

We usually performed the US-guided sclerotherapy using 95 %-ethanol in the early stage of our study, although later we preferred to use fluoroscopic-guided sclerotherapy with 50 %-acetic acid. The mean diameter of the renal cysts was determined on the initial US or CT images before the procedures. The volume of each renal cyst was calculated from images obtained before and after the treatment using the method described by Lin et al. (2005).

$$ V = \left( {l \times w \times d} \right) \times 0.523, $$

where *l*, *w*, and *d* are the geometric length, width, and depth of the cyst, respectively. The procedure was performed in all patients on an in-patient basis. The volume of the sclerosant used was 10–40 % of the calculated volume of the cyst. The maximum volume of the sclerosant was 75 and 100 mL for acetic acid and ethanol, respectively. All patients were monitored during the procedures, and no conscious sedation was required. Patients were placed in a prone position, and the location of the renal cyst was confirmed on US. After appropriate selection of the entry site, we used the Seldinger technique with an 8.5-Fr pigtail catheter. After opacification of the cyst so as to rule out any communication with the renal parenchyma, collecting system or perirenal space, a 50 %-acetic acid or 95 %-ethanol solution was injected via the 8.5-F catheter following aspiration of the fluid from the renal cyst. After clamping of the catheter, the patient was rolled into supine, prone, and lateral decubitus positions at 5-min interval in order to increase the contact between all surfaces of the cysts and the sclerosant. The type and amount of the sclerosant were determined according to the eight operators’ preference.

In the ethanol group, the sclerosant was then evacuated through the pigtail catheter and the catheter was left open for natural drainage following sclerotherapy. If the drainage was more than 10 mL per day, a second sclerotherapy session was performed the following day. The catheter was removed when the amount of daily drainage was <10 mL and when the cavity was seen to be collapsed on US. If the drainage was more than 10 mL per day, an additional sclerotherapy session was performed the following day. In the acetic acid group, the sclerosant was evacuated through the pigtail catheter and the catheter was removed at the time of sclerotherapy as there was only a single sclerotherapy session was according to the protocol for acetic-acid sclerotherapy.

The renal cyst volumes calculated on last follow-up US or CT images were compared with those calculated before sclerotherapy. Comparison of the treatment effects was evaluated by two end-points, i.e. the volume reduction rate (overall regression rate) and the response to the sclerosant. The volume-reduction rate was defined as ([calculated initial volume − calculated post-treatment volume]/calculated initial volume) × 100. The response to the sclerosant was classified as complete regression (<5 % of the initial volume), partial regression (≥5 to <50 % of the initial volume) or no regression (≥50 % of the initial volume). The number of treatment sessions was also compared, and we evaluated the linear association between the initial calculated volume of the cyst and the volume-reduction rate in each group. The patients’ medical records were also reviewed for procedure-related complications.

### Statistical analysis

The categorical variables were summarized by numbers and percentages and the numeric variables, by their mean ± SD (standard deviation). Differences in patients’ demographics were compared across the subgroups using the Chi square test or Fisher’s exact test for categorical variables and the independent t test or the Mann–Whitney U test for numeric variables, as appropriate. Correlations were tested using the non-parametric Spearman’s rank correlation coefficient. p values <0.05 were considered significant. All statistical analyses were performed using SPSS21.0 (SPSS, Inc., Chicago, IL, USA).

## Results

According to the patients’ baseline characteristics, there was no significant difference inpatient age, sex, cyst location (right or left), and the follow-up period after sclerotherapy between the acetic acid and the ethanol groups (Table 1). Cytological examinations were negative for neoplastic cells in all patients.

The mean initial diameter of the renal cysts was 6.6 cm (range 2.9–12.3 cm) in the acetic acid group and 7.0 cm (range 4.0–15.3 cm) in the ethanol group. The mean calculated initial volume of the renal cysts in the pre-treatment imaging study was 194.9 ± 211.2 ml in the 50 %-acetic acid group and 217.9 ± 270.3 ml in the 95 %-ethanol group (Table 1), and there was no statistically significant difference between the two groups (p = 0.116). The used volume of acetic acid was 31.34 ± 17.2 mL (mean ± SD; range 4–75), while the used volume of ethanol was an average of 53.5 ± 31.2 mL (mean ± SD; range 12–100).

Following the treatment, the calculated volume reduction rate was 94.1 ± 7.6 % in the 50 %-acetic acid group and 94.7 ± 11.7 % in the 95 %-ethanol group. No statistical difference was observed between the two groups (p = 0.071). The rates of complete regression, partial regression, and no regression were 57.1 % (n = 24), 42.9 % (n = 18), and 0 %, respectively, for the acetic acid group, and 70.5 (n = 31), 25.0 (n = 11), and 4.5 % (n = 2), respectively, for the ethanol group. No statistical difference was observed between the two groups (p = 0.088) (Table 2).

**Table 2:** Difference in the clinical outcomes and treatment session between the patient groups

Volume reduction	Group		p value
	Acetic acid (n = 42)	Ethanol (n = 44)	
Volume reduction rate (%) (mean ± SD)	94.1 ± 7.6	94.7 ± 11.7	0.088
CR (>95 %)	24 (57.1)	31 (70.5)	
PR (>50 to ≤95 %)	18 (42.9)	11 (25.0)	
NR (≤50 %)	0 (0.0)	2 (4.5)	
Number of treatment sessions (mean ± SD)	1.10 ± 0.30	1.80 ± 0.79	<0.001
1	39 (92.9)	20 (45.5)	<0.001
2	3 (7.1)	16 (36.4)	
3	0 (0.0)	7 (15.9)	
4	0 (0.0)	1 (2.3)	

*CR* complete regression, *PR* partial regression, *NR* no regression

For the volume reduction rate, the total number of procedures was 45 in the 42 patients in the acetic acid group and 77 in the 44 patients in the ethanol group. For the acetic acid group, although the protocol consisted of only a single session of sclerotherapy, two sessions were performed according to the operators’ decisions in three patients. In two of them, sclerotherapy was stopped due to mild flank pain after injection of 5–6 mL of acetic acid during the first treatment session. The second session was successfully performed the next day without any event. In the other patient, two sessions at a 1-day interval were planned and performed due to the operator’s concern regarding incomplete sclerotherapy due to the large volume (570 mL) of the cyst. Compared to the acetic acid group, the ethanol group patients had higher number of sessions (1.10 ± 0.30 for the acetic acid group, 1.80 ± 0.79 for the ethanol group, p < 0.001) (Table 2). It was also determined that there was no significant linear association between the initial calculated volume of the cyst and the volume reduction rate (Spearman’s r = −0.119, p = 0.453 in the acetic acid group; r = 0.217, p = 0.156 in the ethanol group).

Mild flank pain was a minor complication that occurred in four patients in the acetic acid group and in three patients in the ethanol group during sclerosant injection into the cysts. However, there were no major complications in either group. In addition, no additional complications occurred either on follow-up clinical evaluations or during radiologic evaluations.

## Discussion

Although there is controversy regarding the best way to treat simple renal cysts using sclerotherapy (Egilmez et al. 2007), alcohol is the most commonly used sclerosing material for cyst ablation (Amar and Das 1984; Mohsen and Gomha 2005; Ohnishi et al. 1994; Cho et al. 2008; Seo et al. 2000; Lin et al. 2005; Akinci et al. 2005; Germani et al. 2010). 95 %-ethanol is readily available, is inexpensive, and is very slow to penetrate the fibrous capsule of a cyst, thus allowing application of the alcohol and its removal before the renal parenchyma is affected (Bean 1981). However, in addition to several potential alcohol-related complications, cyst recurrence has been reported in more than 30 % of patients and repeated procedures were required in order to compensate for the decreased effectiveness (Kim et al. 2004; Seo et al. 2000; Hanna and Dahniya 1996).

Acetic acid has a stronger cytotoxic effect than ethanol and produces coagulation necrosis (Ohnishi et al. 1994). Acetic acid received Food and Drug Administration (FDA) approval for variety of indications (Won et al. 2004). It is also an economical and safe sclerosant (Seo et al. 2000). Ohnishi et al. (1998) showed that acetic acid injection is superior to percutaneous ethanol injection in terms of both survival and local recurrence rates for small hepatocellular carcinoma in a prospective randomized study. Won et al. (2004) assessed the efficacy of percutaneous acetic-acid sclerotherapy for the management of lymphangiomas. They performed the sclerotherapy procedures with using fluoroscopy guidance for 12 lymphangiomas. In that study, the average reduction of the cyst volume was 93 % at the end of the first year and the cysts completely disappeared in 17 (17.5 %) patients. In their study, complete lymphangioma resolution was obtained in eight patients (66.7 %), good resolution (>50 % reduction) was found in three (25 %), and poor resolution (<50 % reduction) was seen in one (8.3 %). And except for one, all of these patients underwent single-session sclerotherapy.

The first study comparing the effect of acetic acid with ethanol when used for sclerotherapy of a simple renal cyst was performed by Seo et al. (2000). In their study, the remaining cysts were examined 4 months after sclerotherapy using acetic acid. The volume of these cysts was found to be one-half that of the ethanol group. Additionally, the acetic acid group had a larger number of cysts that had regressed to <10 % of the initial volume than the ethanol group. They performed procedures once for each cyst in both groups and concluded that acetic acid is more effective as a sclerosant and induces faster regression of a renal cyst than does ethanol. They suggested that if the concentration of acetic acid was not decreased to <30 %, it was destructive to the epithelium of the renal cyst.

In a study by Cho et al. (2008), they performed one treatment in the acetic-acid group and as many as two treatments in the ethanol group. Their study showed that the rates of complete remission and partial remission were 90.6 and 9.4 %, respectively, for the acetic acid group, and 60 and 30 %, respectively, for the ethanol group, and. The success rate, i.e. complete remission and partial remission was found to be significantly higher in the acetic-acid group (100.0 %) than in the ethanol group (90 %). In our study, the acetic-acid group showed an equivalent sclerosing effect for simple renal cysts with ethanol in terms of both the overall and classified regression rates.

While in our study the number of treatment sessions of the 50 %-acetic-acid group was significantly fewer than that of the 95 %-ethanol group (p < 0.001), it is one of our study’s limitations to have included three patients who underwent two sessions of acetic-acid sclerotherapy contrary to the protocol of single acetic-acid sclerotherapy. There was no patient who underwent more than two sessions in the acetic-acid group, whereas eight patients (18.2 %) underwent more than two sessions in the ethanol group. A small number of treatment sessions may be an important advantage as multiple sessions are associated with a risk for infection, patient discomfort, and high cost (Zerem et al. 2008).

Our study has other limitations. First, as the patient evaluation was retrospective, there was an unavoidable selection bias. Second, because the type and amount of the sclerosant used to perform the treatment were selected according to the operator’s familiarity and preference, variable amounts of sclerosants were used. Various concentrations of the sclerosants may also have effected the treatment results.

## Conclusions

In conclusion, acetic acid seems to have equivalent sclerosing effects on simple renal cysts compared with those of ethanol despites of fewer treatment sessions.
